# Associations between Community Cohesion and Subjective Wellbeing of the Elderly in Guangzhou, China—A Cross-Sectional Study Based on the Structural Equation Model

**DOI:** 10.3390/ijerph18030953

**Published:** 2021-01-22

**Authors:** Shulin Lai, Yuquan Zhou, Yuan Yuan

**Affiliations:** 1 School of Geography and Planning, Sun Yat-sen University, Guangzhou 510275, China; laishl3@mail2.sysu.edu.cn; 2 Guangzhou Urban Planning & Design Survey Research Institute, Guangzhou 510060, China; 3 Department of City and Regional Planning, College of Environmental Design, University of California, Berkeley, CA 94720, USA

**Keywords:** community cohesion, subjective well-being, elderly, structural equation model, Guangzhou

## Abstract

Population aging has become one of the most prominent population trends in China and worldwide. Given the retirement and physical limitation of the elderly, the neighborhood has gradually become the center of their daily lives and communication. Community cohesion plays an essential role in improving the elderly’s subjective wellbeing. However, most present studies on the concept and relationship between different dimensions of community cohesion are mainly in western countries. Meanwhile, most of the studies on the relationship between community cohesion and subjective wellbeing only focused on one aspect of community cohesion such as community interaction. To address this research gap, this study sampled 20 communities in Guangzhou, conducted a questionnaire survey on 969 elderly people, and explored the relationship between four aspects of community cohesion (community interaction, environmental satisfaction, belonging, and participation) and their associations with subjective wellbeing using the Structural Equation Model (SEM). In addition, we performed multi-group analysis to study the association differences among older individuals in communities with different socioeconomic types. We found that: (1) The conceptual relationship between different aspects of community cohesion among older adults is significant; (2) Community environmental satisfaction, interaction, and belonging associate with the elderly’s subjective wellbeing, whereas there is no significant association between community participation and subjective wellbeing; (3) Mental health is an important mediating factor connecting community cohesion and subjective wellbeing, whereas physical health is not. (4) The association pattern of older adults in communities with different socio-economic status are identical, whereas the association strengths are different. In high Socio-Economic Status Index (SESI) communities (communities where older adults with relatively high socioeconomic attributes gather, such as high income and education level), community belonging and participation are significantly associated with community environmental satisfaction and interaction, respectively. In low SESI communities (communities in which older adults with relatively low socioeconomic attributes gather, such as low income and education level), community interaction, belonging, and participation considerably link to community environmental satisfaction, interaction, and belonging, respectively. Regarding the association between community cohesion and subjective wellbeing, community interaction has stronger linkage with the elderly’s subjective wellbeing of in high-SESI aging community than low-SESI aging community. While community environmental satisfaction has stronger association with the elderly’s subjective wellbeing of the elderly in low-SESI aging community than high-SESI aging community. Therefore, it is sensible for community planning to focus on community environment improvement and vibrant community activities organization.

## 1. Introduction

Since the 1950s, aging has gradually become a worldwide population phenomenon. According to the United Nations, it is estimated that by 2050, there will be more older adults (60 years old and above) than adolescents and youth aged 10–24 in the world, and about 80% of the elderly in the world will live in developing countries [1]. Furthermore, the population in China is aging much faster than many other countries [2]. According to the data from the National Bureau of Statistics in China, by the end of 2019, the number of older adults reached 253 million, accounting for 18.1% of its total population [3]. The rapid population aging has entailed a series of challenges to China’s social and economic development and become a concerning issue. Regarding the daily life of the elderly, due to retirement and physical limitations, they spend most of their time in and around their residences [4]. Therefore, family and community relationship networks have become the primary source of their daily recreational activities and social life [5]. Given that the distance between the elderly and their families limits the communication between them, the neighborhood has also become an important place of their daily life and communication.

### 1.1. Conceptualization and Dimensions of Community Cohesion

The literature shows that the concepts of social cohesion and community cohesion are neither clearly differentiated nor uniformly defined [6]. In this study, the concept of community is considered as the neighborhood where residents live and have local networks of interpersonal ties [7], and community cohesion is considered as social cohesion within the setting of older adults’ residential community or neighborhood [6]. The concept of community cohesion covers a rich variety of aspects such as social integration, community identity, and support. Single and multi-dimension methods to assess social cohesion are widely used in existing research. As for the single index measurement, the degree of social interaction is usually the key indicator to define community cohesion [8,9]. However, social cohesion embraces a broader concept such as social capital [10] and therefore, defining it only by social interaction is likely to be incomprehensive. On the one hand, social capital, an evolving and rich concept defined as features of social organizations, such as networks, norms, and trust, facilitates action and cooperation for mutual benefit and promotes social cohesion [11,12]. On the other hand, social cohesion benefits social capital [13]. In addition, social capital is also linked to the degree of welfare and income inequality [14], and norm, as a form of social capital, supports residents in the community with a sense of safety and educational achievement [15].

Faced with the above complex nature of social cohesion, researchers adopted multi-dimension measurement to understand community cohesion better. Kearns et al. established a social cohesion framework, which includes five aspects: belonging and identity, social networks and capital, common values and civil culture, social order and control, and social solidarity and disparity reductions in the rich and the poor [16], which were adopted in multiple studies [17,18,19]. In addition to the five dimensions mentioned above, Colic-Peisker considered feelings of attachment and belonging, participation, services, neighborly help, and neighborhood built environment for community cohesion [6]. Smith put forward a typology of neighborhood cohesion, which includes four dimensions: personal identification, the use of local facilities, social interaction, and consensus on common values [20]. Yuqi Liu et al. operationalized neighborhood cohesion into three aspects: community interaction, participation, and attachment, taking residential environment satisfaction into consideration [21]. Although no unified academic definition of community or social cohesion exists, studies present that community cohesion has various dimensions, which mainly include neighborhood interaction, sense of belonging and attachment, participation, the physical environment of the neighborhood, and so on. Therefore, this study adopts a multi-dimension measurement and builds a community cohesion system by using four indicators: “community interaction” referring to social interaction among residents living in the same community [18,22], representing community social network and social capital, “community belonging” referring to emotional bonds between residents and their community [23,24,25], representing the sense of community attachment and identity, “community environmental satisfaction” referring to satisfaction of neighborhood environment, representing the environmental conditions of the community, and “community participation” referring to the participation in community activities, representing community involvement. 

### 1.2. Interrelationship between Four Dimensions of Community Cohesion

**Figure 1:**
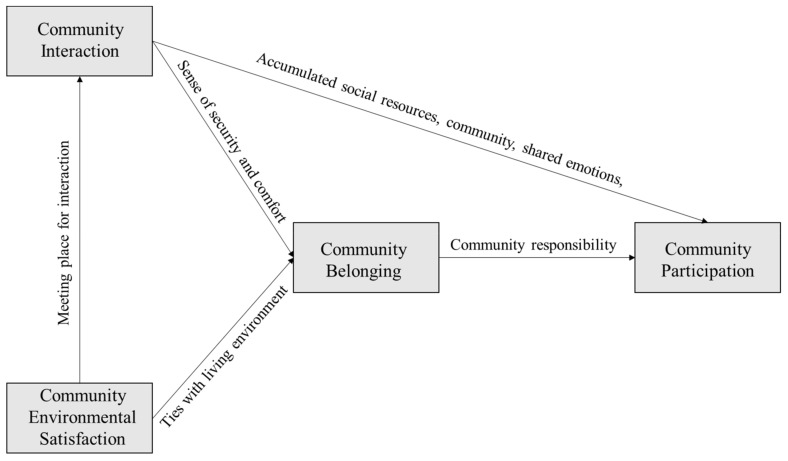
The interrelationship between four dimensions of community cohesion.

The relationship between the four indicators of community cohesion is shown in Figure 1. On the one hand, community interaction positively relates with community belonging given that residents who frequently communicate with their neighbors can obtain a good sense of security and comfort, and therefore tend to have a strong sense of attachment [18,22,23,26,27,28]. On the other hand, community environmental satisfaction positively associates with community interaction and belonging [16,17]. As for community participation, frequent community interactions can help individuals in the community accumulate social resources, develop a sense of familiarity with the community environment, and share emotions with community members, and thereby positively links to their community participation in community affairs and activities [29,30]. Meanwhile, when residents have a sense of belonging, they will have a sense of community responsibility and participate in community affairs and activities [17,22]. Therefore, the community environmental satisfaction, attachment, interaction, and participation are closely related to one another [17,21].

### 1.3. Relationship between Community Cohesion and Individuals’ Subjective Wellbeing

**Figure 2:**
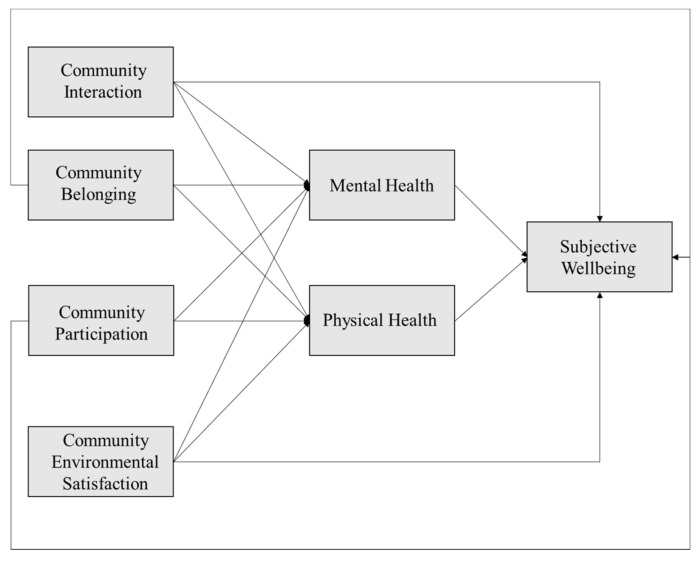
The interrelationship between community cohesion and subjective wellbeing.

As for the association between community cohesion and people’s subjective wellbeing, overall, they are positively related to each other [31], and their relationship is shown in Figure 2. Directly, they are positively linked to each other via the four indicators of community cohesion: community interaction [23,32], participation [33,34,35], belonging [36,37], and environmental satisfaction [38,39,40,41,42]. Indirectly, with community networks providing tangible assistance such as money, care, and transportation, which helps to reduce residents’ mental and physical stress and gives them a safety net [43], community cohesion is associated with individuals’ subjective wellbeing via mental and physical health. First, community cohesion positively associates with residents’ mental health [39]. In a community with strong cohesion, residents can gain an emotional sense of belonging and identity through community interaction and participation, which are beneficial for helping to alleviate residents’ psychological problems [44,45], reducing aggressive behavior and improving residents’ mental health [46]. In addition, community cohesion can potentially alleviate the adverse health effects of insecurity [47], poverty [48], and deprivation [49,50] in the community, which are closely associated with high self-evaluated health conditions and low-depressive symptoms. Second, community cohesion positively links with residents’ physical health. On the one hand, sharing a good community environment, having a strong sense of belonging, and exchanging health information could encourage residents’ health behaviors, such as active physical exercise [51]. On the other hand, community participation, such as activities that includes physical exercise, can create a healthy environment for community members and thus benefit their physical health [52,53]. Third, individuals’ mental health positively links to their physical health status. Mentally healthy individuals have fewer physical limitations in daily life and lower risk of suffering from chronic physical diseases compared with mentally unhealthy individuals [54,55,56,57]. Fourth, residents’ mental and physical health status positively relates to their subjective wellbeing [58]. Positive psychology points out a positive correlation between mental health and subjective wellbeing [59,60]. Concerning physical health, residents who have physical diseases would likely to suffer from subjective distress [61,62,63]. 

### 1.4. Research Gap and Study Goal

However, the concepts and studies of community and social cohesion discussed above are mainly in western countries’ context. Different from communities in western countries where they value the spirit of “autonomy for everyone,” the types of Chinese communities are diverse. Given the changes in China’s social and economic structure, and the reform of the housing system, the cohesion in Chinese communities has been weakened [64]. Meanwhile, social ties not only can bring residents’ access to resources, but can also restrict individual freedoms and bar outsiders from gaining access to the same resources [12], and thus whether community cohesion would benefit residents’ subjective wellbeing, especially in the Chinese context, needs further exploration. Also, current studies on the association between community cohesion and subjective wellbeing often only focus on one dimension of community cohesion or consider all dimensions in isolation. Concurrently, few studies focused on the relationship between community cohesion and elderly’s subjective wellbeing, and the relationship between social capital, community cohesion, income inequality and socio-economic determinants need further research [13,65]. Therefore, this study addresses these research gaps by studying the interrelationship between the four dimensions of the community cohesion and its association with subjective wellbeing among the elderly in Guangzhou, China, while considering the heterogeneity of the mechanism of the aging communities with different socioeconomic status. Therefore, the goal of this research is to propose policy recommendations to promote the older adults’ subjective wellbeing by studying the interrelationship among various dimensions of community cohesion, and its relationship with older adults’ subjective wellbeing.

## 2. Materials and Methods

### 2.1. Hypotheses and Conceptual Model

**Figure 3:**
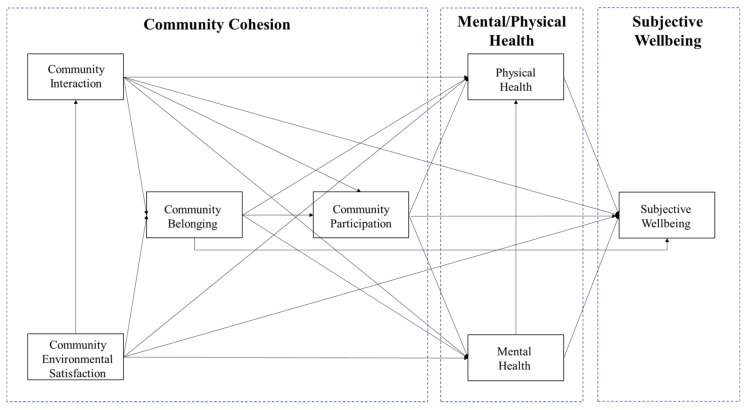
The conceptual model of the study.

The study proposes the following hypotheses on the basis of the above literature review, which is shown in Figure 3 as the conceptual Structural Equation Model (SEM) of the study. First, the relationship between four dimensions of community cohesion (community interaction, belonging, participation, and environmental satisfaction) associate with one another in the following way. Given that the neighborhood environment provides space for social interaction, environmental satisfaction positively links with community interaction. Community environmental satisfaction and community interaction positively associate with community belonging. Meanwhile, community belonging, and interaction positively relate to community participation.

Second, community cohesion could indirectly or directly link with the subject wellbeing of older adults. On the one hand, the four dimensions of community cohesion (community interaction, belonging, participation, and environmental satisfaction) directly and positively associate with older adults’ subject wellbeing. On the other hand, these four dimensions of community cohesion positively relate to older adults’ subjective wellbeing via their mental and physical health.

### 2.2. Study Design, Area and Participants

**Figure 4:**
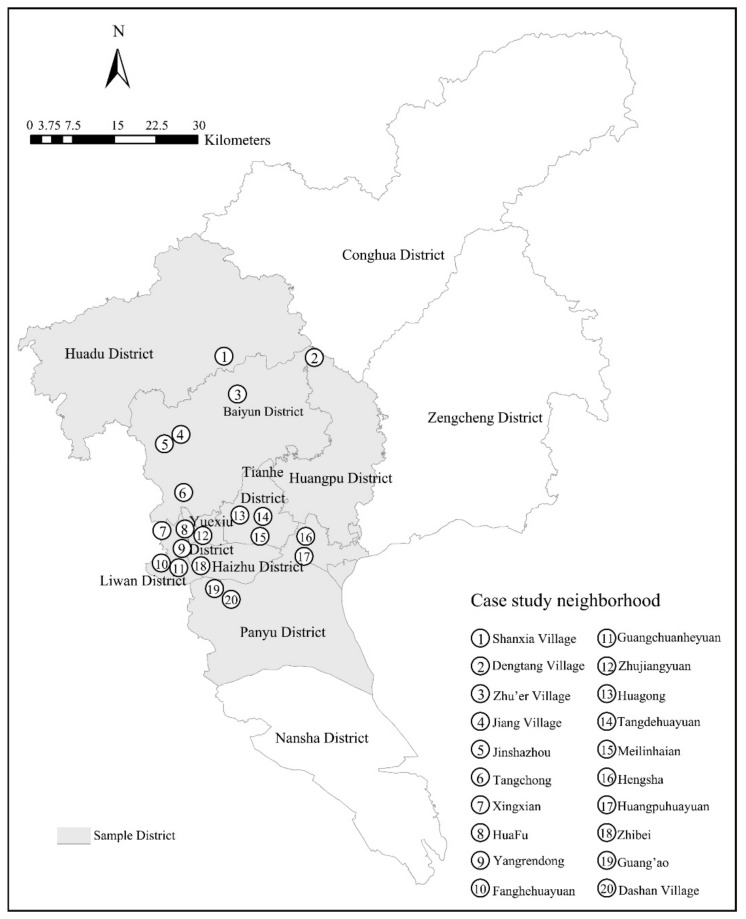
Locations of sampled communities in Guangzhou City, China.

This study’s data is derived from a questionnaire survey conducted on elderly aged 60 and above in Guangzhou City (except Zengcheng, Conghua, Nansha district) from December 2018 to April 2019. On the basis of SPSS statistical analysis software and principal component analysis, combined with the sixth census data, six social aging areas were differentiated (including concentrated distribution areas of older adults in old neighborhoods, in government agencies, enterprises, and institutions, in urban village, and in new development areas of younger generation, and scattered distribution area of retired elderly in education and scientific research units, and mixed population distribution area). A total of 18 streets (jiedao) with the highest scores of main relevant factors among these six social aging areas were selected. The selected communities are of high aging rate within these 18 streets and cover six housing types, including institutional, affordable, historic, rural self-built, commercial, and urban village housing (Figure 4). With the number of questionnaires in each community based on the percentage of its elderly’s population, a multi-stage stratified probability proportionate to population size sampling technique (PPS) was applied to select participants. A total of 969 valid questionnaire surveys of randomly selected elderly aged 60 and older who lived in the selected neighborhood in Guangzhou for over six months were conducted via in-person interviews. This study was reviewed and approved by the School of Geography and Planning, Sun Yat-sen University, and participants provided their written informed consent with the interview survey.

### 2.3. Measurement

Community cohesion comprises interaction, belonging, participation, and environmental satisfaction in a community, within the radius of older adults’ residential communities. Community interaction and belonging are defined by asking each senior to which extent that they agree with the statements “I know many people in the community” and “I belong to this community,” respectively. Using the quintile Likert scale, the responses of “Strongly disagree,” “Disagree,” “Not decided,” “Agree,” and “Strongly agree” and are coded into 1 to 5, respectively. Community participation is measured by the response to “How often did you participate in community activities in the past 12 months?” “Never participated,” “Seldom,” and “Often” were coded into 1 to 3, respectively.

Community environmental satisfaction includes transportation, shopping, medical, housekeeping maintenance, service and payment, environmental sanitation, public security, greenery and overall satisfaction. Measured by quintile Likert scale, the five categories of responses are from “very satisfied” to “very dissatisfied” and are coded into 5 to 1, respectively. Cronbach’s alpha = 0.885 and Kaiser–Meyer–Olkin (KMO) = 0.880 indicate high reliability and validity, which is suitable for the model.

Although some existing studies have adopted other classical health-related tools such as geriatric assessment to evaluate elderly’s health status comprehensively [66,67], this study focuses on the subjective wellbeing of elderly’s in the communities and thus adopts a questionnaire survey to understand elderly’s’ subjective wellbeing and its mediating factors. Subjective wellbeing is measured by asking each senior to which extent they agree with the statements, “I think I am happy.”. “Strongly Disagree” to “Strongly agree” are coded into 1 to 5, respectively. Physical health status comprises to what degree the respondents agree with the ten statements: “I seem to get sick easier than others,” “I have poor health condition,” “Feels hard to do heavy exercise activities (such as running, playing, lifting weights, and so on.),” “Feels hard to do moderate exercise activities (such as lifting tables, cleaning rooms, doing gymnastics, and so on.),” “Feel hard to climb the stairs,” “Feels hard to bend and kneel,” “Feels hard to walk for about 20 min,” “Feels hard to bathe and dress yourself,” “Has your body been in pain (such as headache, chest tightness, nausea, and so on.) in the past four weeks?,” “Has the physical pain affected your work and housework in the past for weeks?”. The response from “Strongly Disagree” to “Strongly agree” are coded into 5 to 1, respectively. Concerning mental health, seven statements, including “I feel calm,” “I feel good and happy,” “I can concentrate on doing the things,” “I don’t feel stressed,” “I am not nervous,” “I don’t feel downcast,” “I feel energetic” were asked, and responses from “Strongly agree” to “Strongly Disagree” are coded from 5–1, respectively. 

The potential moderators are the socio-economic indicators of the elderly, including: gender (male = 0, female = 1); age (continuous variable, year); registered residence status, (registered residence is the same with living address = 0, household registration is different from living address = 1; highest education level (elementary school diploma and below = 1, junior high school diploma = 2, high school diploma = 3, college degree = 4, bachelor’s degree = 5, postgraduate and above = 6); Household per capita monthly income (continuous variable, yuan); individual estimated monthly income (continuous variable, yuan); length of residence (continuous variable, year); housing property rights (self-own housing = 1, children’s housing = 2, rental housing = 3).

### 2.4. Analysis Method

Studies on the association between community cohesion and individuals’ subjective wellbeing in Western countries mostly adopted multiple quantitative models, such as ordinary logistic regression and hierarchical linear model. To further test the logical interrelationship between dimensions of community cohesion and its association with elderly’s subjective wellbeing (Figure 3) fits the elderly population in China, this study adopted the Structural Equation Model (SEM) [68] in Amos based on maximum likelihood estimates. SEM is a directional statistical model that allows researchers to explore associations between one or more variables, which can be either factors or measured variables, by calculating multiple regression analyses of factors [69].

The reliability analysis in SPSS was adopted to evaluate the reliability of the survey result. Cronbach’s alpha coefficients of community environmental satisfaction, physical, and mental health are 0.885, 0.901, and 0.933, respectively, indicating good reliability. Pertaining to validity, the KMO value of the selected data is 0.894, suggesting good validity by passing the Bartlett sphericity test at 99.9% confidence level. 

### 2.5. High and Low SESI Communities Classification

To analyze the association difference between community cohesion and the elderly’s subjective wellbeing in communities with different socio-economic status, K-means clustering method is adopted to analyze the socio-economic status (SES) of the elderly [70,71,72], adopting variables, including registered residence status (hukou), education level, household per capita monthly income, individual estimate monthly income, and housing property rights. Consequently, 969 elderly were categorized into two groups: high and low-SES elderly. With the SESI of 20 sampled communities is calculated (Equation (1)), ten communities with the highest index are defined as high-SESI aging communities, and the rest are defined as low-SESI aging communities.

SESI_i_ = X_1i_ × 2 + X_2i_ × 1 (1)

where i is one of the 20 communities studied in the article. The elderly with high-SES and the elderly with low-SES are coded into 2 and 1, respectively. X_1i_ refers to the percentage of the elderly with high-SES in the community i, X_2i_ refers to the percentage of the elderly with low-SES in community i. 

## 3. Results

### 3.1. Descriptive Statistics

Table 1 shows the characteristics of the studied variables. The average age of all respondents is 70 years old, and 43.0% of them are female. Most of the respondents are locally registered residents (69.0%), have senior high school education level and below (93.2%), and live in self-owned housing (63.6%). The average individual estimate monthly income is 4,531.8 yuan, which is higher than the household per capita monthly income (2970.2 yuan).

Regarding community cohesion, community belonging has the highest average score (4.04), whereas community environmental satisfaction (3.98), community interaction (3.72) rank second and third, respectively, which reflects the quality of the community environment to a certain extent. Within all the items of community environmental satisfaction, the elderly has the highest satisfaction with transportation and shopping conditions (both are 4.16) and the lowest in environmental sanitation (3.77). Overall, the frequency of community participation is relatively low, with 44.0% and 39.0% of the elderly “never participated” or “occasionally participated” in community-organized activities, respectively. 

As for the subject wellbeing level, with 80.6% of the elderly strongly agree or agree with the statement “I think I am happy,” and only 1.1% of the elderly strongly disagree with the statement, the average score is 4.05.

The average physical and mental health scores of the respondents are 3.41 and 3.95, respectively. On the one hand, it reflects that the mental health of the elderly is better than their physical health. On the other hand, it may be that the elderly are concerned about their health status and have high physical health expectations. Concurrently, with a high standard deviation value, the physical health status of the elderly is polarized.

### 3.2. Model Fit and Results

**Figure 5:**
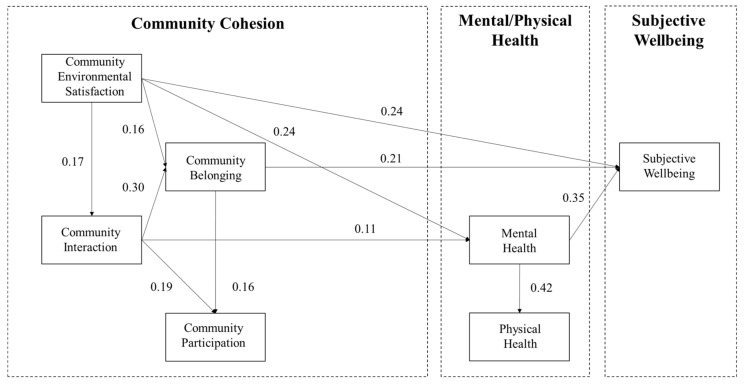
Modified SEM and results.

With the insignificant path removed and after model modifications according to the index MI and t values suggested by the Amos software, the absolute fitness index of the model (RMSEA = 0.050, GFI = 0.922, AGFI = 0.905), simple fit index (PGFI = 0.754, PNFI = 0.824) and value-added fitness index (IFI = 0.954, CFI = 0.954, TLI = 0.954) all meet the requirements of model fit, showing a good fit to the data. The summarized results are shown in Figure 5 and Table 2.

Concerning the interrelationship between four dimensions of community cohesion, all the theoretical associations are positive and statistically significant at 99.9% confidence interval, which means that all proposed conceptual associations are proved to be true in this empirical study. Community environmental satisfaction positively associates with community belonging and interaction, whereas community interaction positively associates with community belonging and participation. Furthermore, community belonging positively links with community participation.

No significant association exists between social cohesion and the elderly’s physical health status. However, the community environmental satisfaction and community interaction significantly and positively associate with the elderly’s mental health status at 99.9% confidence interval. Meanwhile, the elderly’s mental health status significantly and positively links with their physical health and subjective wellbeing. 

Looking at the overall association between community cohesion and the elderly’s subjective wellbeing, community environmental satisfaction, belonging, and interaction directly or indirectly associate with the elderly’s subjective wellbeing. The overall standardized association estimate between community environmental satisfaction and subjective wellbeing is 0.370, and the direct association (0.235) is greater than the indirect association (0.135). The indirect associations are realized via community interaction, community belonging, and mental health. Community interaction indirectly associates with subjective wellbeing (0.102), which is achieved via community belonging and mental health, and community belonging directly links with subjective wellbeing (0.213) (Table 3).

### 3.3. Multigroup Analysis

Based on the classification of Equation (1), the result shows that high-SESI aging communities concentrate in historic, institutional, and commercial housing neighborhoods and are mostly self-owned housing (69.7%). Older adults living in high-SESI communities are mainly local residents (75.0%) with higher educational and income levels (individual estimate monthly income: 5740.8 yuan, and household per capita monthly income 3514 yuan). In contrast, low-SESI communities are mostly in urban villages, affordable housing, and rural self-built housing neighborhoods and have a relatively high percentage of rental housing (27.0%). Elderlies living in low-SESI communities have a relatively large percentage of non-local residents (38.3%) with lower educational and income levels (individual estimate monthly income: 2869.4 yuan, and household per capita monthly income: 2222.5 yuan). As for the four indicators of community cohesion, high-SESI communities have significantly higher scores in community environmental satisfaction (average 4.06) than low-SESI communities (average 3.87), but have lower scores in community interaction, participation, and belonging (3.50, 4.02, 1.66) than low-SESI communities (3.76, 4.06, 1.78). (Table 4)

Multigroup analysis is performed using the model with highest fitness (CMIN/DF = 2.498, RMSEA = 0.039, PGFI = 0.731, PNFI = 0.829, TLI=0.904, IFI = 0.943, CFI = 0.942) among unconstrained, measurement weights restricted, structural weights restricted, structural covariances restricted, structural residuals restricted, and measurement residuals restricted model.

**Figure 6:**
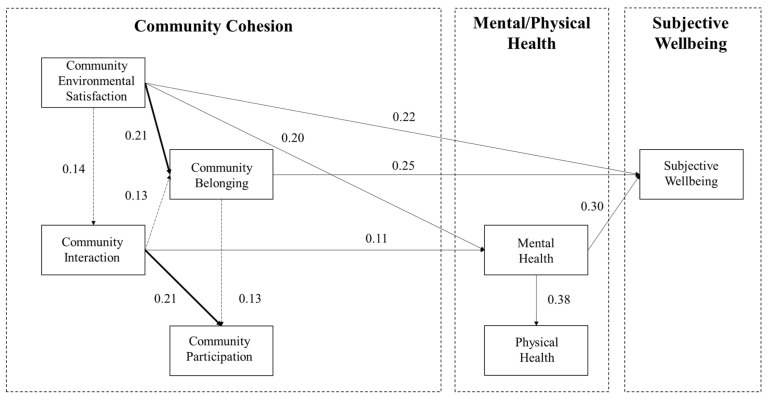
Modified SEM results of High-SESI Community Group. The paths that are considered significantly different have a critical ratio greater than 1.96.

**Figure 7:**
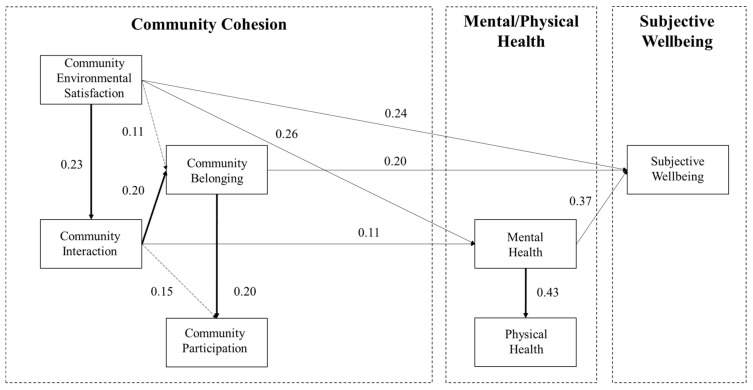
Modified SEM results of Low-SESI Community Group. The paths that are considered significantly different have a critical ratio greater than 1.96.

Although the associations paths between community cohesion and the elderly’s subjective wellbeing are the same in high- and low-SESI communities, some parameters are statistically significantly different (Figure 6 and Figure 7, & Table 5). When the critical ratio between parameters of the corresponding paths in two groups >1.96 (at the 95% confidence interval or higher), the two corresponding paths were seen as significantly different [73]. Pertaining to the interrelationship between the four dimensions of community cohesion, with low environmental satisfaction in low-SESI communities, the improvement of community satisfaction greatly associates with the increasing frequency of community interaction than in high-SESI aging communities (0.227 > 0.143). On the one hand, the community belonging of the low-SESI communities is more associated with community interaction (0.305 > 0.297), whereas the community belonging of the high-SESI communities is more associated with community environmental satisfaction (0.254 > 0.181). On the other hand, the community participation of the low-SESI communities is more associated with community belonging (0.199 > 0.132), whereas the community participation of the high-SESI communities is more associated with social interaction (0.251 > 0.208).

As for the overall association, community environmental satisfaction of low-SESI communities has greater direct and indirect linkage with community interaction, participation, mental and physical health, and subjective wellbeing than high-SESI communities. Meanwhile, the community interaction of high-SESI communities has a greater direct and indirect association to community participation, mental health, and subjective wellbeing than low-SESI communities. Ultimately, the elderly in high-SESI communities are more sensitive to community interactions than those who are in low-SESI communities. Older adults who are in low-SESI communities are more sensitive to environmental satisfaction than those who are in high-SESI communities. (Table 5)

## 4. Discussion

By conducting the questionnaire survey, SEM analysis, K-means clustering, and multigroup analysis, this study contributes to the current knowledge on this research theme in the following dimensions. First, this paper innovatively analyzed the systematic interrelationship between various dimensions of community cohesion and its association with individuals’ subjective wellbeing in the Chinese context. Second, this study especially focused on the elderly, which enriched aging relevant research and provides creative perspective for age-friendly community planning. Third, the study analyzed the association difference between high-SESI and low-SESI aging communities. Consistent with existing studies, this study verified the interrelationship between four indicators of community cohesion (community environmental satisfaction, interaction, and belonging), and its association with individuals’ subjective wellbeing, as well as its mediating associations via mental health, which reaffirmed the practical value of the research frameworks of Western-developed countries in China. Beyond the existing studies, this study also makes additional findings in the Chinese context. First, relatively speaking, community participation does not have a strong association with the elderly’s mental health, physical health, and subjective wellbeing. Second, perhaps due to the intermediary effect of family care, physical health does not have a noticeable association with the subjective well-being of the elderly. 

### 4.1. Interrelationship between Four Dimensions of Community Cohesion

First, community environmental satisfaction positively relates to community interaction. A good and satisfying community environment creates a suitable communication space for the elderly. For example, adequate greenspace creates comfortable communication spaces, and good public security conditions create a safe and reliable communication atmosphere, thus encouraging the community interaction behaviors of the elderly in the community [73]. Meanwhile, different socio-economic groups of people have a different perception of the environment. For example, women are more sensitive than men in the way they perceive the surrounding environment, and they see the role of trees more strongly as building good social interactions than men [74], and older adults, especially those who with mobility difficulties, tend to spend time in community parks to contact with nature and connect social relations with others [75,76]. As for the elderly in Guangzhou, from the interviews and surveys, we learned that they normally exercise with sports equipment, chat, play cards and Mahjong in community open spaces with acquaintances and friends. Second, community environmental satisfaction positively associates with community belonging. The higher the satisfaction the elderly have with the community environment, the stronger the self-confidence in the community and the higher the sense of community belonging, because people have attachment not only with people, but also with their immediate living environment [77]. Third, the higher the degree of community interaction, the stronger perception of community belonging, such as the sense of security, comfort and order individuals feel in the community [22,26,28,78]. Fourth, community interaction and belonging positively relate to community participation. Community belonging can promote a sense of community responsibility among the elderly, which encourages them to participate in community activities and decisions [17,26], and the frequent interactions among community residents can enhance mutual trust, and thus encouraging them to participate in community activities [29].

### 4.2. Association between Community Cohesion and the Elderly’s Health Status

Consistent with our hypothesis based on existing literature, community environmental satisfaction and community interaction of the elderly positively relate to their mental health. The result shows that community cohesion is conducive to residents’ mental health by reducing their daily life stress, which aligns with the conclusion of an empirical study in the United States [79]. The mechanism behind the linkage between community environmental satisfaction and the elderly’s mental health is probably, as Jacobs believed that good public security could reduce residents’ fear of crime, increase their sense of security, assuring to let their children go out and play in the community [80]. Therefore, it benefits the elderly’s mental health by lifting their pressure of caring for their grandchildren and relieving their stress. Meanwhile, community interaction among the elderly, such as chatting, can help release their stress [81], ease their tension [82], and positively affects their perception of self-aging status and the evaluation of self-worth [83]. These conclusions are consistent with the existing empirical studies which apply to broader community environmental elements and age group. For example, environmental satisfaction with commercial facilities, sanitary conditions, public green spaces, and recreational facilities positively relates to residents’ mental health [84]. Meanwhile, high community environmental satisfaction can give residents a sense of community belonging and support, thereby even reducing the psychological pressure caused by poor housing conditions [85,86]. As a place of interaction and communication, green space satisfaction will facilitate community support and residents’ activities, and thus reduce residents’ loneliness [87], while being around nature could relieve stress and fatigue, which balances mental state and significantly and positively associates mental health [88]. Especially for older adults, community interaction positively relates to the elderly’s perception of self-aging status and the evaluation of self-worth and is vital to their mental health [83].

However, this study fails to find a significant linkage between the elderly’s community belonging, participation, and mental health, indicating that the elderly’s mental health status is not dependent on their sense of community belonging and community participation. A possible explanation for that is that for the older generation, participation may be an issue of habit and social pressure [89], and thus its linkage between older adults’ mental and physical wellbeing may not be significant. As for community belonging, though a number of existing studies found a positive association between it and individuals’ mental health [84,90,91], these studies are not in the context of older adults in Guangzhou, which is an inclusive and vibrant migrant city in China.

Besides, this study has not found a significant relationship between the elderly’s community cohesion (community environmental satisfaction, interaction, belonging, and participation) and their physical health, which does not agree with some of the existing study results. The existing research shows that a good community environment, such as walkability, can significantly improve the level of physical exercise and reduce the morbidity rate of chronic diseases, such as being overweight and obesity among residents [92,93]. Meanwhile, some other researchers concluded that the effect of physical exercise and activities on physical health is not significant [93,94]. A possible explanation of the insignificant association in this study is that there may be other mediating indicators in the linkages, such as physical exercise. However, this study focuses on the association between community cohesion and older adults’ subjective wellbeing, considering physical health as a mediating factor. Therefore, with a considerable number of existing studies analyzing the relationship between community cohesion and individuals’ health status, and the intention of maintaining the reliability of the SEM from over complexity, this study only explored the direct association between community cohesion and older adults’ physical health without considering other mediating factors such as physical exercise. Regarding social interaction, the possible reason for the insignificant association may be that instead of physical exercise, chatting and playing cards are the main interaction methods for the elderly in Guangzhou, which increase their probability of sedentary and do not positively impact their physical health [95,96]. As for community belonging, existing studies found the association between it and physical health is different by gender, and therefore, it is possible that this conclusion does not apply to the elderly group [97]. Meanwhile, a significant association is neither found between the elderly’s community participation and their physical health nor mental health. A potential reason behind it is that the definition of community participation in this study mainly focuses on community activities and based on the information older adults provided in the interview, most of the activities held for the elderly often focus on painting and calligraphy competitions and anti-fraud safety lectures, which have trivial impacts on their physical activity and wellbeing. In contrast, the community participation in most of the existing studies focuses on the political aspects and represents residents’ participation in the community decision-making process, which has a positive effect on their well-being [36,37].

### 4.3. Association between Community Cohesion and Subjective Wellbeing of the Elderly

Overall, community cohesion positively relates to older adults’ subjective wellbeing. A possible explanation behind it is that the older adults’ radius of activities tends to fall within their walking accessible distances due to more prevalence of functional limitations [98,99] and less need for commuting [100,101] compared to other age groups. 

Specifically, community environmental satisfaction associates with the elderly’s subjective wellbeing directly and via community interaction, belonging, and mental health, which is consistent with the existing studies indicating that residents in the communities who have high environmental satisfaction, such as better neighborhood green space, neighborhood security, public service supply, transportation accessibility, and so on tend to have ideal subjective wellbeing [40,41,42,102]. For example, transportation accessibility could promote residents’ subjective wellbeing by shortening travel time [103], and green spaces could promote their subjective wellbeing by reducing air pollution, heat, and noise exposure [104] and provide buffer space for relieving their life pressures [105,106,107]. Meanwhile, community interaction associates with the elderly’s mental health via community belonging and mental health, extending the conclusion of the existing research, which puts forth that community interactions can alleviate the pressure of residents and mitigate the negative effect of stressful events on subjective wellbeing, thereby promoting individuals’ subjective wellbeing [82]. However, no direct relationship is found between community interaction and the elderly’s subjective wellbeing. Probably because not the quantity but quality of community interactions matters, low-quality community interactions have negative effects, such as ineffective help, excessive help, unnecessary help, and so on [108].

Community belonging directly and indirectly associates with the elderly’s subjective wellbeing via mental health. In comparison, physical health has no significant association with the elderly’s subjective wellbeing. One possible explanation is that there may be a mediating linkage between physical health and subjective wellbeing. In addition, we found that self-rated health status of the elderly (1–5, the higher the score, the better the health) and whether they live with their children (0 = Yes, 1 = No) are significantly positively correlated (Pearson correlation coefficient is 0.69 *, *p* = 0.031), that is, the elderly who self-assessed their poor physical condition often live with their children. Hence, they may receive more family care and love, thereby enhancing their happiness.

However, this study does not find an association between community participation and older adults’ subjective wellbeing, which contradicts with the empirical study that found a positive relationship between social participation and subjective well-being among retirees in China [109]. A possible reason is that in this study, participants are of various employment status, and only 68.9% of the participants and retirees. Besides, the scope of social participation in that existing study is broader and included four aspects: frequency of social activities, roles in social activities, working state, and participation in activities of former employing units, while the participation in this study only includes community participation.

### 4.4. Association Linkages between High- and Low-SESI Communities

Overall, most elderly who live in low-SESI communities have a lower income, lower education level, poorer neighborhood environment, higher percentage of migrant residents and higher community interaction, belonging, and participation level than high-SESI communities. Part of the reason behind it is that older adults living in low-SESI communities, especially in rural villages, have strong community attachment, because they have lived in the community since they were born and almost know everyone in the community [110]. Also, the result shows that community environment improvement is positively related to the community cohesion, subjective wellbeing, and mental and physical wellbeing of the elderly in low-SESI communities. Contrarily, most older adults in high-SESI communities have high income, high education level, high community environmental satisfaction, and low community interaction, belonging, and participation level. Because community interaction has a prominent association with community participation, subjective wellbeing, and mental and physical wellbeing in high-SESI communities, and therefore the spillover effects of community interaction in high-SESI communities are weakened. Besides, studies have shown that the migrant population often faces difficulties in housing, medical care, employment, and so on, and therefore their subjective wellbeing is lower than the local population [111], while higher income, higher educational attainment and housing property rights all contribute to have better subjective wellbeing [108,112,113,114,115], indicating that the improvement of social status positively impacts individuals’ subjective wellbeing [116,117].

### 4.5. Strengths and Limitations

This study mainly has two strengths. First, we investigated the interrelationship between four dimensions of community cohesion. Second, we further analyzed the association linkages of the elderly in high and low SESI communities to identify the differences and provide accurate conclusions and suggestions.

Meanwhile, the study has some limitations that could be resolved in future research. First, as a cross-sectional study, it is challenging to infer a causation relationship but reveal the positive associations between studied variables. Second, the single indicator of subject wellbeing and community participation may lead to bias and narrowed definition. If the elderly encounter occasional incidents, the subjective wellbeing evaluation at the time may deviate from their long-term feelings, making it difficult for single-indicator evaluation to reflect the elderly’s long-term subjective wellbeing. In future research, we can refer to “The Satisfaction with Life Scale” [118] and “Positive Affect and Negative Affect Scale” [119,120] to establish and refine a subjective wellbeing indicator system that can evaluate the emotional state of the elderly over a period of time and take the political decision-making perspective of community participation into account. Third, subjective questionnaire surveys may cause difficulty in the objective application of research conclusions and this study has not considered the reciprocal relationships between variables. Therefore, future research could deeply explore the relationship between subjective judgments and objective existence to apply them well in urban and rural planning practice and investigate the reciprocal relationships between community cohesion and individuals’ wellbeing.

## 5. Conclusions

By establishing a theoretical SEM on the basis of existing studies, the modified model was used to explore the interrelationships between four dimensions of community cohesion and its linkages between community and the subjective wellbeing of the elderly in 20 residential communities in Guangzhou, China. Furthermore, K-means clustering method was used to categorize the SES of the elderly, and a multigroup analysis was conducted to analyze the association difference among elderly in high- and low-SESI aging communities. The results of this study extend the knowledge on the research theme in the Chinese context in the following aspects. First, in China, the linkage between community participation with the elderly’s mental health, physical health, and subjective wellbeing is not prominent. Second, physical health does not play a significant role in elderly’s subjective wellbeing. Probably because different from the elderly in Western countries, who tend to live in relatively smaller households, the elderly in China rely on strong family ties, which play an important role in maintaining their subjective wellbeing [11,121,122]. Third, as for the interrelationship between community cohesion’s four indicators, community environmental satisfaction and interaction of the elderly in high-SESI aging communities have a significant linkage with their community belonging and participation, respectively, whereas community interaction, belonging, and environmental satisfaction of the elderly in low-SESI aging communities has a strong association with their community belonging, participation, and interaction, respectively. Overall, the elderly’s subjective wellbeing is more associated with community interaction and belonging in high-SESI communities, while it is more related to community environmental satisfaction in low-SESI communities. Based on our conclusions, to improve the elderly’s community cohesion, subjective welling, mental and physical health, planners are recommended to focus on community environment planning, especially for low-SESI aging communities. Community environmental improvement could be realized by diverse party participation. For example, the improvement of transportation, shopping, and medical infrastructure should be led by the government. Meanwhile, members of the community should participate and play critical roles in community environmental sanitation, public security, and greening improvement. Community organizers could also hold creative and vibrant community activities to promote elderly’s community participation.

## Figures and Tables

**Table 1:** Variable statistical summary.

Variables	Proportion/Mean (Standard Deviation)
*Population characteristics (total population = 969)*	
Gender (%)	
Male	43.0%
Female	57.0%
Registered residence status	
Local registered resident	69.0%
Nonlocal registered resident	31.0%
Highest education level	
Elementary school diploma and below	41.4%
Junior high school diploma	28.0%
Senior high school diploma	23.8%
College degree	4.1%
Bachelor’s degree	2.6%
Post-graduate degree and above	0.1%
Average Age	70
Individual estimate monthly income	4531.8
Household per capita monthly income	2970.2
Housing property rights	
Self-owned housing	63.6%
Children’s housing	12.9%
Rental housing	23.5%
*Community cohesion*	
Community interaction: “I think that I know many people in the community” (1–5)	3.72 (0.971)
Community belonging: “I belong to this community” (1–5)	4.04 (0.882)
Community participation: “How often did you participate in community activities in the past 12 months?” (1–5)	1.73 (0.733)
Average community environmental satisfaction (1–5)	3.98 (0.614)
Transportation satisfaction (1–5)	4.16 (0.726)
Shopping satisfaction (1–5)	4.16 (0.700)
Medical satisfaction (1–5)	3.98 (0.856)
Housekeeping maintenance satisfaction (1–5)	3.87 (0.823)
Service and payment satisfaction (1–5)	3.98 (0.798)
Environmental sanitation satisfaction (1–5)	3.77 (0.993)
Public security satisfaction (1–5)	3.97 (0.831)
Greenery satisfaction (1–5)	3.94 (0.834)
*Subjective wellbeing*	
“I think I am happy.”	4.05 (0.821)
*Potential Mediators*	
Physical Health (1–5)	3.41 (0.892)
Feels hard to do heavy exercise activities (such as running, playing, lifting weights, etc.) (1–5)	2.84 (1.220)
Feels hard to do moderate exercise activities (such as lifting tables, cleaning rooms, doing gymnastics, etc.) (1–5)	3.36 (1.176)
Feels hard to climb the stairs (1–5)	3.17 (1.229)
Feels hard to bend and kneel (1–5)	3.30 (1.209)
Feels hard to walk for about 20 min (1–5)	3.670(1.236)
Feels hard to bathe and dress yourself (1–5)	4.000 (0.924)
Has your body been in pain (such as headache, chest tightness, nausea, etc.) in the past four weeks? (1–5)	3.37 (1.236)
Has the physical pain affected your work and housework in the past for weeks? (1–5)	3.545 (1.180)
Mental Health (1–5)	3.95 (0.766)
I feel calm (1–5)	4.05 (0.800)
I feel good and happy (1–5)	4.02 (0.873)
I can concentrate on the things that I am doing (1–5)	3.99(0.850)
I don’t feel stressed (1–5)	3.88 (1.039)
I am not nervous (1–5)	3.95 (0.931)
I don’t feel downcast and nothing can cheer me up (1–5)	3.94 (0.921)
I feel energetic (1–5)	3.80 (0.931)

**Table 2:** Standardized estimates and the significance index of modified associations in Structural Equation Model (SEM).

Association between	Community Environmental Satisfaction	Community Interaction	Community Belonging	Community Participation	Mental Health	Physical Health
Community interaction	0.171 ***	—	—	—	—	—
Community belonging	0.162 ***	0.300 ***	—	—	—	—
Community participation	—	0.186 ***	0.156 ***	—	—	—
Mental health	0.239 ***	0.111 ***	—	—	—	—
Physical health	—	—	—	—	0.417 ***	—
Subjective wellbeing	0.235 ***	—	0.213 ***	—	0.346 ***	—

*** means significant at 99.9% confidence interval; — means non-exist association linkage or deleted association linkage due to SEM requirement.

**Table 3:** Overall, direct, and indirect association between the studied variables based on the SEM.

	Association Type	Community Environmental Satisfaction	Community Interaction	Community Belonging	Community Participation	Mental Health	Physical Health
Community interaction	Overall association	0.171	—	—	—	—	—
	Direct association	0.171	—	—	—	—	—
	Indirect association	0.000	—	—	—	—	—
Community belonging	Overall association	0.214	0.300	—	—	—	—
	Direct association	0.162	0.300	—	—	—	—
	Indirect association	0.052	0.000	—	—	—	—
Community participation	Overall association	0.065	0.233	0.156	—	—	—
	Direct association	0.000	0.186	0.156	—	—	—
	Indirect association	0.065	0.047	0.000	—	—	—
Mental health	Overall association	0.258	0.111	—	—	—	—
	Direct association	0.239	0.111	—	—	—	—
	Indirect association	0.019	0.000	—	—	—	—
Physical health	Overall association	0.107	0.046	—	—	0.417	—
	Direct association	0.000	0.000	—	—	0.417	—
	Indirect association	0.107	0.046	—	—	0.000	
Subjective wellbeing	Overall association	0.370	0.102	0.213	—	0.346	—
	Direct association	0.235	0.000	0.213	—	0.346	—
	Direct association	0.135	0.102	0.000	—	0.000	—

— means non-exist association linkage or deleted association linkage due to SEM requirement.

**Table 4:** Socio-economic and community cohesion indicators of the elderly in high/low-SESI aging communities.

Variables	High-SESI Aging Communities (Percentage/Mean)	Low-SESI Aging Communities (Percentage/Mean)
Registered residence status (hukou)		
Local registered resident	75.0%	62.7%
Nonlocal registered resident	25.0%	38.3%
Highest education level		
Elementary school diploma and below	30.3%	56.6%
Junior high school diploma	30.7%	24.3%
Senior high school diploma	29.8%	15.7%
College degree	5.7%	2.0%
Bachelor’s degree	3.4%	1.5%
Post-graduate degree and above	0.2%	0.0%
Individual estimate monthly income	5740.8	2869.4
Household per capita monthly income	3514.0	2222.5
Housing property rights		
Self-owned housing	69.7%	55.1%
Children’s housing	9.3%	17.9%
Rental housing	21.0%	27.0%
Community cohesion		
Community interaction: “I think that I know many people in the community” (1–5)	3.50	3.76
Community belonging: “I belong to this community” (1–5)	4.02	4.06
Community participation: “How often did you participate in community activities in the past 12 months?” (1–5)	1.66	1.78
Average Community environmental satisfaction (1–5)	4.06	3.87
Transportation satisfaction (1–5)	4.22	4.07
Shopping satisfaction (1–5)	4.18	4.13
Medical satisfaction (1–5)	4.06	3.87
Housekeeping maintenance satisfaction (1–5)	4.00	3.70
Service and payment satisfaction (1–5)	4.04	3.90
Environmental sanitation satisfaction (1–5)	3.85	3.66
Public security satisfaction (1–5)	4.09	3.80
Greenery satisfaction (1–5)	4.04	3.80

**Table 5:** Overall, direct, and indirect association in high- and low-SESI communities.

	Association Type	Community Type	Community Environmental Satisfaction	Community Interaction	Community Belonging	Community Participation	Mental Health	Physical Health
Community interaction	Overall association	High-SESI	0.143	—	—	—	—	—
		Low-SESI	0.227	—	—	—	—	—
	Direct association	High-SESI	0.143	—	—	—	—	—
		Low-SESI	0.227	—	—	—	—	—
	Indirect association	High-SESI	0.000	—	—	—	—	—
		Low-SESI	0.000	—	—	—	—	—
Community belonging	Overall association	High-SESI	**0.254**	0.297	—	—	—	—
		Low-SESI	0.181	**0.305**	—	—	—	—
	Direct association	High-SESI	0.212	0.297	—	—	—	—
		Low-SESI	0.112	0.305	—	—	—	—
	Indirect association	High-SESI	0.042	0.000	—	—	—	—
		Low-SESI	0.069	0.000	—	—	—	—
Community participation	Overall association	High-SESI	0.064	**0.251**	0.132	—	—	—
		Low-SESI	**0.069**	0.208	**0.199**	—	—	—
	Direct association	High-SESI	0.000	0.212	0.132	—	—	—
		Low-SESI	0.000	0.147	0.199	—	—	—
	Indirect association	High-SESI	0.064	0.039	0.000	—	—	—
		Low-SESI	0.069	0.061	0.000	—	—	—
Mental health	Overall association	High-SESI	0.218	**0.113**	—	—	—	—
		Low-SESI	**0.280**	0.108	—	—	—	—
	Direct association	High-SESI	0.201	0.113	—	—	—	—
		Low-SESI	0.255	0.108	—	—	—	—
	Indirect association	High-SESI	0.017	0.000	—	—	—	—
		Low-SESI	0.025	0.000	—	—	—	—
Physical health	Overall association	High-SESI	0.082	0.043	—	—	0.379	—
		Low-SESI	**0.122**	**0.047**	—	—	0.435	—
	Direct association	High-SESI	0.000	0.000	—	—	0.379	—
		Low-SESI	0.000	0.000	—	—	**0.435**	—
	Indirect association	High-SESI	0.082	0.043	—	—	0.000	
		Low-SESI	0.122	0.047	—	—	0.000	
Subjective wellbeing	Overall association	High-SESI	0.347	**0.107**	**0.246**	—	0.302	—
		Low-SESI	**0.378**	0.102	0.203	—	**0.369**	—
	Direct association	High-SESI	0.218	0.000	0.246	—	0.302	—
		Low-SESI	0.238	0.000	0.203	—	0.369	—
	Direct association	High-SESI	0.129	0.107	0.000	—	0.000	—
		Low-SESI	0.140	0.102	0.000	—	0.000	—

Bold figures refer to the parameters are higher than the corresponding parameters in the other community group. — means non-exist association linkage or deleted association linkage due to SEM requirement.

## Data Availability

The data are not publicly available due to institutional copyright and privacy issues. Requests to access the datasets should be directed to Yuan Yuan, yuanyuan@mail.sysu.edu.cn.
